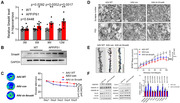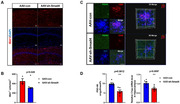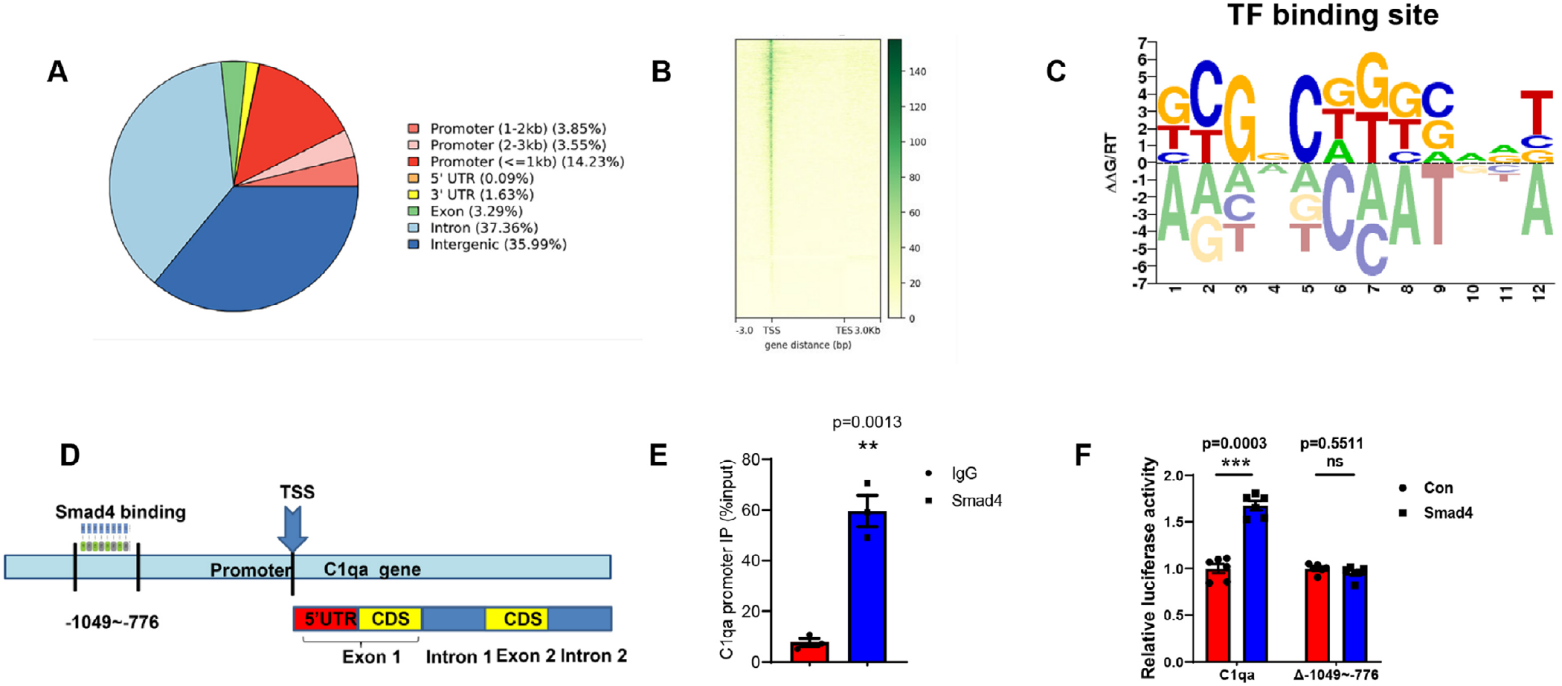# Inhibition of Smad4 improves the cognitive function of APP/PS1 mice by targeting the level of c1qa

**DOI:** 10.1002/alz.084896

**Published:** 2025-01-03

**Authors:** Jianwei Ge, Benyan Luo

**Affiliations:** ^1^ First Affiliated Hospital, School of Medicine, Zhejiang University, Hangzhou, Zhejiang China; ^2^ First Affiliated Hospital, Zhejiang University, Hangzhou, Zhejiang China

## Abstract

**Background:**

Synaptic plasticity impairment plays a critical role in the pathogenesis of Alzheimer’s disease (AD), Smad4, a central intracellular signal transmission mediator of transmission of transforming growth factor‐β (TGF‐β) signaling, plays a pivotal role in many biological processes, including cell differentiation, migration, apoptosis and tumorigenesis. Emerging evidence has demonstrated that Smad4 is also involved in the pathogenesis of AD. Once TGF‐β signaling is stimulated, Smad4 interaction with Sp1 and Smad3 induces the transcriptional activation of APP. Smad4 physically binds with TGF‐β1‐induced anti‐apoptotic factor (TIAF1) and prevents TIAF1 self‐aggregation, which reduces production of Aβ and amyloid fibrils.

**Method:**

The adeno‐associated virus knocking down Smad4 (AAV‐sh‐Smad4) and the corresponding control were respectively injected into the bilateral hippocampus of 6‐month‐old APP/PS1 mice using a stereotaxic apparatus. Open filed tests, novel object recognition tests, Morris water maze (MWM) tests and contextual fear conditioning tests were applied to test the cognitive function of APP/PS1 mice. Real‐time quantitative PCR and western blot was used to measure the expression of Smad4. Patch clamp technique was used to record the LTP and fEPSP of the hippocampal CA1 slices. Golgi staining and transmission electron microscope were applied to evaluate the number and structure of synapses. Luciferase activity assay, cleavage under targets and tagmentation assay and chromatin immunoprecipitation were applied to discover and verify the potential target c1qa of Smad4.

**Result:**

The level of Smad4 was significantly increased in the hippocampus of APP/PS1 mice. Smad4 inhibition alleviates memory deficits in 6‐month‐old APP/PS1 mice. Smad4 inhibition significantly increased the number of synapses and thickness of PSD in APP/PS1 mice. Furthermore, Smad4 inhibition increased the dendritic spine density and the percentage of mushroom spines in APP/PS1 mice as demonstrated by Golgi staining. Cut&Tag assay was performed to explore the potential targets of Smad4, and it showed that Smad4 negatively affected the expression of c1qa by directly binding to the ‐1049 to ‐776bp of the c1qa promoter with CHIP and Luciferase activity assay. Immunofluorescence showed inhibition of Smad4 effectively reduce the phagocytosis of synapses by microglia.

**Conclusion:**

Inhibition of Smad4 improves the cognitive function and synaptic plasticity of APP/PS1 mice by targeting the level of c1qa.